# Translational research—the need of a new bioethics approach

**DOI:** 10.1186/s12967-016-0773-4

**Published:** 2016-01-15

**Authors:** Sorin Hostiuc, Alin Moldoveanu, Maria-Iuliana Dascălu, Runar Unnthorsson, Ómar I. Jóhannesson, Ioan Marcus

**Affiliations:** Department of Legal Medicine and Bioethics, Carol Davila University, Bucharest, Romania; National Institute of Legal Medicine, Bucharest, Romania; Faculty of Automatic Control and Computers, Polytechnic University of Bucharest, Bucharest, Romania; Department of Engineering in Foreign Languages, Polytechnic University of Bucharest, Bucharest, Romania; Faculty of Industrial Engineering, Mechanical Engineering and Computer Science, University of Iceland, Reykjavik, Iceland; Department of Psychology, University of Iceland, Reykjavik, Iceland; Department of Pathophysiology, Faculty of Veterinary Medicine, University of Agricultural Sciences and Veterinary Medicine, Cluj-Napoca, Romania; Sos.Vitan Barzesti 9, Sector 4, 042122 Bucharest, Romania

**Keywords:** Translational bioethics, Phase analysis, Data transfer

## Abstract

Translational research tries to apply findings from basic science to enhance human health and well-being. Many phases of the translational research may include non-medical tasks (information technology, engineering, nanotechnology, biochemistry, animal research, economy, sociology, psychology, politics, and so on). Using common bioethics principles to these areas might sometimes be not feasible, or even impossible. However, the whole process must respect some fundamental, moral principles. The purpose of this paper is to argument the need for a different approach to the morality in translational bioethics, and to suggest some directions that might be followed when constructing such a bioethics. We will show that a new approach is needed and present a few ethical issues that are specific to the translational research.

## Background

Translational science is a novel concept, whose main purpose is to categorize practical, outcome-oriented research [1]. In healthcare, translational research can be viewed as research on human specimens, whose findings may inform basic science research and lead to a transfer of the results towards clinical therapeutics and novel healthcare policies; its definition seems however to be an ever evolving phenomenon; if initially it referred to the bench-to-bedside enterprise of using information from basic sciences to produce new treatment alternatives for patients [2], nowadays it is defined by a process that starts with fundamental research (genes, molecular processes, biochemical pathways) and ends at a macro level (social healthcare, access to healthcare, access to education, and so on) [3]. Moreover, some authors considered that translational process should not start with fundamental research, as this approach rarely succeeds, but rather from clinical medicine—the so-called bedside to bench to bedside approach. A classical example to show the usefulness of this approach is represented by the development of balloon angioplasty by Gruentzig [4]. A more recent example is represented by T cell therapy—T cells have various roles in cell-mediated immunity, being able, among others to differentiate between healthy and abnormal (including neoplastic) cells, and is involved in HIV infection. This clinical knowledge has recently been brought to the “bench” by a research team lead by dr. Marson, who, by editing the genome of human T (CD4+) cells using a CRISPR/Cas9 ribonucleoprotein delivery method, caused up to 40 % of the cells to lose high level cell surface expression of CXCR4 [5], which could open new therapeutic possibilities in oncology, autoimmunity or infectious diseases.

**Fig. 1:**
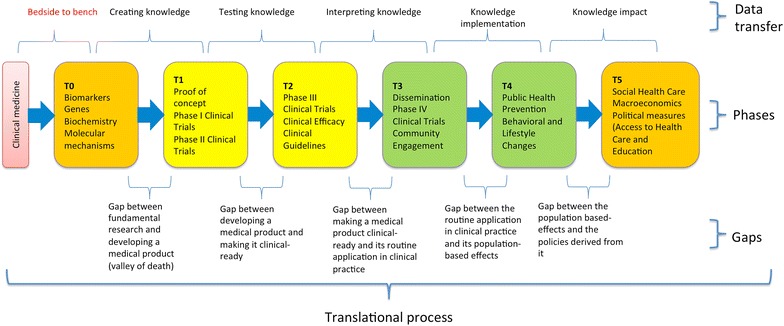
Translational research—from fundamental research to developing policies (based on information retrieved from [61] and [3])

For the purposes of this article we will try to define the translational process as containing three main elements: phases, gaps, and data transfer; therefore, when analysing ethical issues determined by translational research we will consider them as appertaining to either an element of the translational process (phase, gap, data transfer), or the process of translational research itself (see Fig. 1—modified after [3, 6, 7], and Table 1 for some examples of ethical issues depending on the element of the translational process).

**Table 1:** Examples of ethical issues depending on the translational process elements

Translational process	Ethical issues	Examples
Phases	Mainly derived from research ethics—animal bioethics, research of clinical trials, etc.	Informed consent in clinical trials for the development of assistive devices Social injustice and discrimination caused by using neural prostheses to enhance neurocognitive processes [62]
Gaps	Ethics of resource allocation	Allocation of public research grants for either “safer” research, with high chances of success but limited healthcare impact versus unconventional research with smaller chances of success but greater potential healthcare impact
Data transfer	Research misconduct, data confidentiality	Sharing of data obtained from neural sensory input that is used in modelling software medical solutions [63, 64]
Process of translational research as a whole	Social justice, translational approach of risks, data sharing and protection	Conducting medical trials in developing countries

Initially translational research was considered as containing two main phases: T1—bench-to-bedside, in which new discoveries from the laboratory could be translated towards clinical research (proof of concept, phase I and II clinical trials) and T2, in which these applications were translated in clinical practice (phase III clinical trials, studies of clinical efficacy and development of clinical guidelines). Recently the process was further developed by the addition of other stages; therefore, the most recent models contain either four or six translational phases [3, 6] and may or may not include the bedside-to-bench approach. For the purpose of this article, we will use the six-phase approach shown in Fig. 1. The six-phase approach considers that translational research starts with fundamental research and ends with developing and implementing social/economical policies determined by the results obtained in the process.

Between each adjacent set of phases is a gap, or roadblock, which refers to the lack of funding/support needed for the progression to the next [8]. The most important gap is between T0 and T1, called *valley of death* because most fundamental research studies do not go beyond it (therefore to not enter the clinical phases). According to Pienta these translational gaps are dependent upon the successful management of four main risks: scientific, intellectual property, regulatory, and market [8].

In the translation process, the transfer of information from one study to the next one characterizes data transfer [8]. These transfers occur either within phases or between phases (usually adjacent, but not always). The type of data transferred depends on the location of the transfer on the translational research process. For example the data that is transferred in within the first two phases is usually newly created data, which stays at the basis of the whole translational research process. The information created in this stage is structured in a way that can be easily transferred toward clinical research. For example, suppose that an investigator discovers a new heart receptor for angiotensin in cell cultures. This information, which is newly created, is transferred to another preclinical investigator, who might test its clinical consequences on animal models. The created data from this study is then transferred to a pathologist who will test the effect of the receptor on the pathological human heart. The data is then transferred to another researcher who might be involved in developing a drug that could be helpful for that specific cardiac pathology. All this transfer of newly created data happens in Phase 0. Next, the information could be transferred to a clinical investigator who will try a Phase 0 study on humans with the above-mentioned drug. Each data transfer is directed toward a certain group of individuals (that can be anything from a small group of researchers to a major population group). The size of the group that receives the transmitted data increases in size with the advancement of translational process. For example, the target group for transferring newly created knowledge (during fundamental research) is represented by investigators involved in basic and subsequently clinical research, whose number is usually very small. When going from Phase 2 to Phase 3 the knowledge will be transferred to clinicians, patients, epidemiologists, and so on, a much larger target group. Data transfer is usually done step by step in translational research. However, there are instances in which the process can skip one or more phases. For example, detecting a gene that is often associated with cancer might lead directly to dissemination of the information, or even public health initiatives, without going through T1 and T2 phases.

According to the Merriam-Webster dictionary, bioethics is defined as a discipline dealing with ethical implications of biological research and applications, especially in medicine. This definition is very broad, as it includes biological research (medical research, animal research, environment research), and medical practice. However, it is limited when trying to analyse recent developments in medical research, in which non-biological components are starting to have a bigger importance—information technology (IT) development, nanotechnologies, engineering, and so on (see Table 2 for an example). These developments lead to two important questions that must be answered before defining the scope of translational bioethics: (1) Should we analyse translational research ethics in direct relation to the steps/phases, or should we develop a more general set of bioethical guidelines guiding the entire translational research process?, and (2) Should we consider translational research as a subtype of biomedical research, or should we develop a new set of bioethical tools specifically directed to the often transdisciplinary characteristics of translational research?

**Table 2:** Example of translational research—assistive device helping blind people to see with the aid of audio and haptic inputs

Phase	Aims	Tasks
T0	Identifying the way sensory inputs overlay at the brain level	1. *Neuroscience* *2. Prospective/observational studies*
T1	Developing audio and haptic sensors Developing software to aid the translation of audio and haptic sensors to visual inputs or aids	3. Proof of concept 4. IT development
T2	Studying the usefulness of the device Measuring neural/sensory inputs/outputs	5. Using beta testers in closed/open environments (NOT research subjects) 6. *Possible medical research (bio*-*feedback)*
T3	Disseminating the results to patients and clinicians	7. *Certification of the device (analysis of the risk*–*benefit ratio)—might be needed a clinical trial* 8. Increase awareness about the technology (marketing) 9. Promote the technology to stakeholders 10. *Comparing the effectiveness of the technology with others at a population level, identifying possible limitations/adverse reactions*
T4	Changing public health policies and behaviors beyond the effects of the treatment	11. Shift from intervention to prevention (public health campaigns, recommendations about lifestyle changes based on the results obtained from the previous phases)
T5	Social policies	12. Decreasing societal disparities, improving access to healthcare for underdeveloped communities, interacting with cities in order to maximize the input

The completion of each preceding task is needed for the development of the next task

Italic emphasized tasks are biomedical; non-emphasized tasks have a significant non-biomedical component

The purpose of this article is to analyse whether a bioethics approach based on current, healthcare centred principles, is enough to allow a comprehensive model for the morality of translational research.

## Should we analyse translational research in direct relation to the steps/phases, or should we develop a more general set of bioethical guidelines guiding the entire translational research process?

Most articles dealing with translational bioethics limit their analysis to one or few steps of the translational research process. For example Kimmelman [9] analysed the biases generated by risk assessment of clinical trials by the Ethics Review Boards. Shapiro and Layde analysed case scenarios in different phases of translational research, in order to identify the critical role of bioethics in each stage of clinical and translational research [10]. Bærøe considered that translational ethics includes two main discussion topics: (1) the ethics of translational biomedical research, in which we must analyse the various practical scenarios derived from the translational research, and (2) the internal gap between theory and practice within the field of ethics itself [11]. Hyun and Kimmelman made a phase oriented ethical analysis in translational gene transfer research [12]. The first topic is a typical step-by-step analysis of research ethics issues, while the second is a more general issue, recognized by some authors in the last years, related to the development of a rift between theoretical bioethics and its implementation in medical practice [13, 14]. The translational research process contains however not only phases but also gaps between phases, and transfer of knowledge in which the results are translated from one phase to another, translation that could also pose ethical issues. To illustrate this, we will present a situation generated by translating information from Phases 1 and 2 to Phase 5 from Table 3, in which is detailed the translational process involving in vitro fertilization with sperm procured from the deceased [15]. Recently a series of articles was published that supported the idea of using a presumed consent model for this technique meaning that, in the absence of a refusal from the deceased husband before death, the wife could use the posthumously harvested sperm for in vitro fertilization [16, 17]. Tremellen and Savulescu [16] used a predominantly theoretical approach to the argumentation in favour of presumed consent, while Hans [17] based his conclusion on a sociological study that supported this option. The translation presented by both articles (from clinical practice to social health policy) is associated with ethical issues. In order for a theoretical approach to be allowed for developing social policies, it should be based on a set of accepted general moral guidelines and an exhaustive analysis of the issue (including legal, social, economical and anthropological issues). An analysis not based on the above-mentioned issues could lead to wrong conclusions, which implemented in clinical practice might lead to clearly unethical actions. For example, Tremellen and Savulescu, arguing for a need of a presumed consent on the issue, stated that:

**Table 3:** Example of translational research—in vitro fertilization after posthumous sperm procurement

Phases	Aims	Tasks
T0	Identifying characteristics and viability of semen after death	*Sampling postmortem reproductive products*
T1	Testing in real environments	*Case reports, a few hundred cases worldwide—procreative autonomy, consent from the dead, moral status of the embryo, conflicts of interests*
T2		
T3	Disseminating the results to patients and clinicians	*Increased awareness about the technique (scientific journals, mass media)* *Comparing the effectiveness of the technology with other IVF techniques*
T4	Changing public health policies and behaviors beyond the effects of the treatment	*Establishing guidelines regarding IVF after PSP* *Promoting the technique outside classical scenarios (sampling for fertilization other persons than the wife)*
T5	Social policies	Generalizing the concept of presumed consent for PSP Decreases the number of unfertile couples caused by the death of a husband Increases the birth rate Changes in the resource allocation for ARTs

Italic emphasized tasks are biomedical; non-emphasized tasks have a significant non-biomedical component

*In our view, it can be argued that the welfare of the living is a far more important consideration than splitting hairs over degrees of consent and infringement of alleged autonomous rights of a deceased person. It is important to remember that the dead person no longer exists, so at that time deceased cannot have interests or be autonomous. Any ‘respect’ is related to creating policies that ensure that the living now are not harmed or fail to have their autonomous wishes respected—that is satisfied by an opt out system for posthumous conception. Put succinctly, if you don’t want it, sign out now. (page 9)* [16].

Most Civil Codes, at least from Europe, consider that a person, even if it has died, can exert posthumously a series of rights, through testamentary dispositions. This means that the person retains some “autonomous rights”. The Declaration of Geneva (2006) and the World Medical Association (WMA) Code of Ethics (2006) both state that medical confidentiality must be maintained after the patient dies. Medical confidentiality is based, at least partially, on the right to autonomy (to autonomously choose how to share medical information about one self). Most countries consider that organ harvesting from deceased should be based on a previous consent of the donor, or at least on the consent of the family (the opt in variant for organ donation, present in most countries including Germany, Japan, Romania). There are however some counties, especially in Europe, which have a form of presumed consent (opt out) procedure for organ donation (including Austria, Croatia, Spain, Belgium). The type of procedure (opt-in or opt-out) seems to be a very important reason for organ donation, as revealed by the rates from Germany to Austria, two countries with highly similar cultures—in Germany, a country that preferred the opt-in method, the consent rate is around 12 % while in Austria, a country that preferred the opt-out method, the consent rate is almost 100 % [18–21].

Therefore, we cannot really talk, at least in current medical practice, about the absence of autonomy after the death of the patient. In our opinion, using subjective argumentation, not based on current legislative and generally accepted practices in order to support the development of a health-care policy is unethical, as it may be considered a form of manipulation, exercised over stakeholders. This manipulation could lead to the creation of health-care policies that would have to be applied in clinical practice by physicians, who will be put in a position to respect either a fundamental code of ethics or deontology (again, at least in Europe, many countries have Physicians’ Codes of Ethics that rely heavily on the WMA Code of Ethics), or the national health care policies. By not respecting the healthcare policies, they could do something illegal, while by not respecting the deontological/ethical norms of the medical practice they would do something immoral. Hans used a sociological survey that showed an increased support for posthumous sperm procurement [17]. This approach again contradicts fundamental ethical principles in medical practice. The WMA Code of Ethics states: “A physician shall respect the right of the patients.” [22]. Imposing a certain procedure based on statistical analysis (what most of the population might want) contradicts the principle of autonomy in medicine, which basically states that a person should be able to make informed decisions about one-self. For example, most patients would agree to suffer a surgical intervention for peritonitis (so statistically there is an agreement about accepting the procedure if they were the patients needing that intervention); however, if they are conscious, they are still required to give their consent before entering into the surgery, and are allowed to refuse it. Again, the patient status of the deceased is debatable; however, as the medical codes of ethics recognize some autonomy related rights for deceased patients, it is assumed that they have at least, a form of autonomy, that they could very well exercise before dying. So, in order to respect the autonomy of the patient an opt-in alternative to medical procedures after death should be imposed, not an opt-out variant. The patient should be able to make informed decisions about what will happen to him/her after death; if this does not happen, unsubstantiated assumptions about his/hers agreements for various medical procedures should not be allowed, as they are legally and morally problematic (affecting his/hers self-determination).

A very hot topic in translational research, which presents perfectly the intricacies and limits of bioethics in translational research is represented by stem cell research. Tremendous progress is made every year, requiring a permanent redefinition of what is ethically permissible in stem cell research, and what topics remain actual in this field. For example in the 1990s, major issues in this area were focused on obtaining embryonic stem cell research from aborted fetuses or cloning for the creation of embryonic stem cells for transplantation [23–28]. In the next decade the number of articles in this area exploded, a more than tenfold increase being identified on Web of Knowledge; the articles began to deal with more specific aspects of stem cells ethics, including patents, commercialization, creation of adult cell lines, creation of part animal stem cells, etc. [29–37]. From 2011 to 2015 a shift is again seen, this time from embryonic stem cells ethics to adult or induced pluripotent stem cells (IPSC) ethics [38–42]. When working with IPSCs researches do not have to analyse the morality of using embryos, and therefore debates around abortion, moral status of the embryo, commercialization of human embryos, teleology of the human embryo are not relevant. They do have however important ethical issues to deal with. For example IPSCs are known to form teratomas when injected in immunodeficient mice [43, 44]. Therefore, IPCSs may have the potential to generate human cancers when injected to treat various diseases. This could lead to ethical debates regarding the primordiality of beneficence (physicians have a moral duty to help their patients) versus non-maleficence (physicians have a moral obligation not to do harm to their patients). Uncertainty related to the long term benefits or risks is another ethical issue related to IPCS—should physicians recommend such a therapy taking into account that the long term benefits and risks are not fully quantifiable? [42]. Should IPCSs used as an alternate method to gamete donation in assisted reproductive technologies? [45].

As shown in Table 1, translational research has ethical issues related to any of its four main elements (phases, gaps, transfer of knowledge, the process itself). Even if all are potentially equally important, most researchers in this area focused solely on the phase related ethical issues [1, 2, 9–12, 46–54], which, in our opinion, is not enough for a comprehensive ethical analysis of translational research. However, by analysing all ethical related issues associated with all its elements, and taking into account the high variability of translational research, we could easily find ourselves in the position of considering that translational bioethics should deal with most, if not all, imaginable bioethical issues. This approach, even if exhaustive, does not grasp the particularities of the translational research bioethics. Therefore, our opinion is that we should try to analyse more specifically what makes translational research unique from other forms of biomedical research, and to draw a series of general ethical norms with a wide applicability in this field.

## Should we consider translational research as a subtype of biomedical research, or should we develop a new set of bioethical tools specifically directed to the interdisciplinary characteristics of translational research?

Most codes of research ethics require all biomedical research to be carried out under the supervision of a certified physician or other properly qualified health care professional. For example, the Helsinki Declaration, Art 12 states that:

*Medical research involving human subjects must be conducted only by individuals with the appropriate ethics and scientific education, training and qualifications. Research on patients or healthy volunteers requires the supervision of a competent and appropriately qualified physician or other health care professional. (Article 12)* [55].

However, there are instances of translational research in which most of the phases contain non-medical studies/activities. See Table 2 for such an example, in which the researchers are trying to develop an assistive device for visually impaired people by using haptic and audio signals; the translational process include 12 steps, of which only four can be considered as biomedical research. Most of them are either IT development or public health related (social policies, interactions with local administration, and so on). If we consider them to be biomedical research, they should comply to all legal and ethical requirements of biomedical research, which means, among others, obtaining consent from the subjects, obtaining an Ethical Committee approval for the study, supervision by medical personnel, and so on. Let us take an example from Table 2. Suppose that one step of this research is to develop a medical device constructed as a wearable head unit that enables the user to precisely determine the spatial characteristics of sounds. For this, some testers must walk around the lab and be subjected to various sound patterns. The responses of the testers are recorded. By relating this research to the principles from the Helsinki Declaration this activity fits within the purposes of biomedical research: “The primary purpose of medical research involving human subjects is to understand the causes, development and effects of diseases and improve preventive, diagnostic and therapeutic interventions (methods, procedures and treatments)” [55] because its purpose is to develop a therapeutic intervention for visually impaired people. However, the same activity can be considered a development phase for an IT product. How should the researchers consider this activity? If is it considered biomedical research, it must comply with all medical research regulations that would maximize the protection of the testers (physically, ethically and legally), but also increase the costs maybe tenfold. The precautions may be excessive and unnecessary as the risks are minimal and can be safely handled by other means; e.g., work protection, and the testers are competent to make decisions about the precautions needed for the study. If it is considered an IT development phase—instead of a biomedical research—the importance of the risks will be lowered because a risk assessment might not be performed before starting the tests. The issues related to protection and sharing of personal data (e.g., data obtained from the records of the responses of the subjects) might also be minimized. Our opinion is that the problem here is determined by the phase analysis of translational research. By using it we tend to reffer to biomedical/research ethics guidelines even in instances in which they are too strict, in order to apparently maximize the protection for subjects/patients/citizens. However, this apparent strictness affects the efficiency of the phases that are not actually biomedical research, by imposing time consuming and highly expensive tasks. A more holistic approach to bioethics in translational research might be of use for the whole process aimed toward minimizing the risks for subjects, other involved persons, and the society as a whole. This should be associated with a clear definition of what biomedical research is, and of course with imposing additional safeguards for activities that will comply with the definition of biomedical research. For the above example, we could do a risk assessment before the practical phases and develop methods for minimizing/removing the risks by a committee consisting of both investigators with healthcare specialization and non-medical personnel. Afterwards, the non-medical tasks will be handled according to the IT development principles, in full compliance with the risk mitigation methods established in the initial phase.

## Some proposals for general ethical principles guiding translational research

### Consequentialist analysis of the translational research process

Before starting a translational research the investigators should analyse potential effects not only for the subjects’ population, but also on a more general level. Translational research should not be performed only for scientific curiosity or prestige for the researchers but also for the benefit of the society. This statement might be considered as limiting fundamental research, which is not the case. The main purpose of translational research is to translate fundamental research to clinical practice and beyond. Therefore the above principle should only apply to research that is build having in mind a translational process. For example, suppose that an investigator wishes to study a gene involved in hypertension. In order to translate this research into clinical practice he should not think only about results derived from the genetic analysis, but also on potential clinical applications (gene therapy, or developing a drug that interacts with the product of the gene and decreases blood pressure). He must also consider the population groups that may benefit from the research, the potential cost and the location of the clinical studies. He must reason whether a product is actually needed keeping in mind the multitude of antihypertensive drugs and whether such a drug can become a de facto standard in the treatment of hypertensive patients or if it will only be useful for selected subgroups. Furthermore, he must also consider whether the costs associated with the translational research process would not be better spend for another drug, and so on. Such an analysis will allow us to see not only the immediate consequences of the study, but also future developments, and should allow a better analysis of its clinical usefulness. Moreover, this analysis will allow a better management of medical research funds, with a targeted increase of funds for medical issues lacking enough financing (a better resource allocation, therefore maximizing the benefit resulted from the available finances for biomedical research).

### Translational analysis of the risks associated with the research

If we are to analyse the risks for each phase we might either stop the research if the risks are too high, or to continue the research if they are minimal, irrespective of the overall risks that might be caused by that particular research. For the first variant the easiest example comes from oncology. Developing a new drug comes with very high risks for Phase I subjects. If we were to consider only the risks to which the participants are subjected to, we might stop the research, as the risks might be considered too high. However, without the research, and without the data obtained form this step, the morbidity and mortality decrease associated with the use of that particular drug, would not happen. For the second variant, we can imagine a phase II clinical trial in which the drug seems to have significantly better results compared with other drugs with only an insignificant increase of the risks. These risks however might become important (either increases in number or the detection of rare adverse reactions with severe consequences) if we were to use a larger number of subjects, risks that were identified by other studies analysing similar drugs. Therefore, by not taking into account the increase in statistical power that comes with increasing the numbers of subjects/patients and the data from similar pharmacological agents, we minimize the importance of the potential risks associated with the tested drug. Thus, before starting a translational research process, we should do a more exhaustive analysis of the risks associated with it, even if they are only potential, or only suggested by similar studies. The analysis of risk within the translational process should be done using a consequentialist approach, because minimizing the risks for the subjects and even at a population level should be an important objective of translational bioethics.

### Social justice

In order to increase the degree of social justice caused by a particular translational research, there should be a correspondence between where the research is done, by whom, with which finances, on which subjects and for which potential beneficiaries, while protecting at the same time the vulnerable populations (women, children, psychiatry patients, prisoners, people from developing countries, and so on). Suppose for example that an investigator wishes to develop a very potent antihypertensive drug, whose market cost would be 10.000 USD. For such a drug the potential beneficiaries would be, at least in the first instance, people from developed countries. Therefore, it should not be allowed for this type of clinical trial to take place in a developing country, where there would be a very low number of potential beneficiaries. Here we see how the consequentialist analysis of potential applications of translational research would limit the inclusion of subjects from developing countries, based on more objective criteria (impossibility to pay for the medical product). Currently there are a lot of clinical trials conducted in developing countries, which would benefit patients mainly from developed countries, that clearly contradicts the bioethical principle of justice, according to whom, in the widest normative sense, equals should be treated as equals, and un-equals, un-equal. By performing clinical trials in developing countries, for drugs that would be primarily used in developed countries we tend to treat equals (human beings), un-equally (some suffer the risks while other ripe the benefits). Of course that such an approach should not limit the extension of the treatment to other groups that are more vulnerable (vulnerable patients should receive additional benefits compared to non-vulnerable ones [56]).

### Data sharing

Information from initial stages of the translational research process often appears in the headlines, suggesting potential benefits well before the initiation of clinical studies. A substance shown to diminish the number of cancer cells in cell cultures could be presented as a miraculous treatment for cancer. This dissemination could potentially pose risks, as patients with that disease could try on their own those substances, not yet proven to be effective in clinical studies. Therefore, the analysis of potential consequences of published information from initial studies should also be performed in the early stages of translational research. If data considered sensitive would be published in scientific journals, its presentation should emphasize the not-ready-to-market status of the drug, and potential consequences of using it before there are established treatment guidelines. Moreover we should always take into account the risks of improper dissemination of information to the media (see the Wakefield case [57] and its severe consequences regarding vaccination practices [58, 59]. Another risk with data obtained from translational research is its potential use for bioterrorism/military applications. For example, in 2011 researchers from Japan to Netherlands announced the creation of a modified H5N1 influenza virus, which was transmissible through aerosols between ferrets; until that time, the virus was only known to be transmissible through direct contact with the infected animals. H5N1 infects rarely humans, but the fatality rate is around 60 % [60]. Immediately after US intelligence analysts began to assess the potential security risks associated with the publication of those results. One of the most important elements of a scientific paper is reproducibility—other researchers, using the information from a specific article, should be able to recreate the experiment, in order to test its validity. The researchers developing modified flu viruses should present enough data to make their experiments reproducible. However, reproducibility also means that a virologist working for a terrorist organization could be able to reproduce the results. Ethically such a research is not publishable—if the researchers publish it, and the information is reproducible, there is a risk of bioterrorist applications; if the information is not reproducible, the article lacks clarity and its publication could be considered a form of scientific misconduct; if the researchers do not publish the article but perform the experiment, funds are wasted, and it could pose ethical issues regarding resource allocation (especially if the research is publically funded. These risks should also be assessed before drafting the protocol for a translational research, and if the possibility for such misuses is high, we should try to perform an exhaustive risk–benefit ratio analysis for the whole translational research process.

## Conclusions

The ethics of translational research should go beyond the classical topic of research ethics, or medical ethics. It should not only analyse the ethical issues that can be directly derived from the translational phases but also those derived from the gaps between translational phases, transfer of knowledge and the particularities of translational research per se. There are many potential issues appertaining to the bioethics of translational research, of which only a few have been summarized in this article, and that should be reanalyzed within the translational research framework.
